# Vagally Mediated Gut-Brain Relationships in Appetite Control-Insights from Porcine Studies

**DOI:** 10.3390/nu13020467

**Published:** 2021-01-30

**Authors:** Charles-Henri Malbert

**Affiliations:** 1 Aniscan Unit, INRAE, Saint-Gilles, 35590 Paris, France; charles-henri.malbert@inrae.fr; 2 National Academy of Medicine, 75000 Paris, France; 3 Adelaide Medical School, University of Adelaide, Adelaide, SA 5000, Australia

**Keywords:** miniature pig, pig model, functional brain imaging, molecular imaging, vagal afferents, single fiber recording, insulin resistance, GLP-1r, gastric barostat, gastric emptying, scintigraphy

## Abstract

Signals arising from the upper part of the gut are essential for the regulation of food intake, particularly satiation. This information is supplied to the brain partly by vagal nervous afferents. The porcine model, because of its sizeable gyrencephalic brain, omnivorous regimen, and comparative anatomy of the proximal part of the gut to that of humans, has provided several important insights relating to the relevance of vagally mediated gut-brain relationships to the regulation of food intake. Furthermore, its large size combined with the capacity to become obese while overeating a western diet makes it a pivotal addition to existing murine models, especially for translational studies relating to obesity. How gastric, proximal intestinal, and portal information relating to meal arrival and transit are encoded by vagal afferents and their further processing by primary and secondary brain projections are reviewed. Their peripheral and central plasticities in the context of obesity are emphasized. We also present recent insights derived from chronic stimulation of the abdominal vagi with specific reference to the modulation of mesolimbic structures and their role in the restoration of insulin sensitivity in the obese miniature pig model.

## 1. Introduction

Large animal and murine models have contributed to the understanding of vagally mediated gut-brain relationships since the early recognition of the importance of this pathway for appetite control by Iggo in the UK and Mei [1] in France initially in cats [2] and subsequently in sheep [3]. Both pioneered the single fiber recording of mechanical and chemical vagal digestive afferents. Unfortunately, while the vagus is easy to dissect in these species, the carnivorous or the herbivorous regimens were only remotely close to dietary patterns in humans, raising uncertainties about translational relevance. Recently, genetic identification of the functional population of abdominal vagal afferent neurons in a murine model has been published [4]. The screening strategy is fundamentally based on the histological identification of the neuronal ending. However, the available electrophysiological evidence has been unable to associate one neuronal shape with a distinct functional outcome. For example, while intraganglionic laminar endings are certainly mechanosensitive [5], that does not preclude the possibility that other neuronal types are mechanosensitive. An alternative approach to evaluate the vagally driven appetite control is to investigate brain activity directly. Unfortunately, the primary integration site, the dorsal vagal complex (DVC), is challenging to reach with a microelectrode, necessitating irreversible, invasive surgery. Similarly, imaging the DVC is equally demanding in large animal models given the requirement for ultra-high field (7T) fMRI [6] or partial volume correction with nuclear imaging methods [7]. Unlike the primary integration area, secondary structures involved in cortical and sub-cortical projections of vagal neurons are more accessible to evaluation, when quantitative imaging methods are available in a gyrencephalic species, to facilitate translational interpretation. These methods include a 3D digital atlas of the pig [8] together with the co-registered templates [9] and adapted algorithms [10]. The strategies used to investigate appetite directly and indirectly in the pig model are initially reviewed. Meal-related modulation of gastric and intestinal vagal afferents relevant for appetite control are presented. Finally, the appetite-related effects of chronic stimulation of vagal abdominal trunks are described. A particular focus is on data derived from our laboratory.

## 2. Appetite, Satiety, and Their Measurements

Satiety and satiation are the critical players in the control of appetite. Satiety engulfs the many processes occurring between meals triggered by food consumption and it is usually measured in humans by subjective ratings of hunger and fullness, both of which are not feasible in any preclinical animal model [11]. Satiation occurs during a meal and brings the meal to its end while ultimately determining meal size. Perhaps surprisingly, in humans, the method for evaluating satiation is often limited to measurement of the size of a meal given that the approach is frequently more comprehensive in animal models [12]. For example, the refined analysis of the structure of the meal [13] aims to dissect the temporal evolution of satiation with a time resolution close that of the physiological processes.

**Figure 1:**
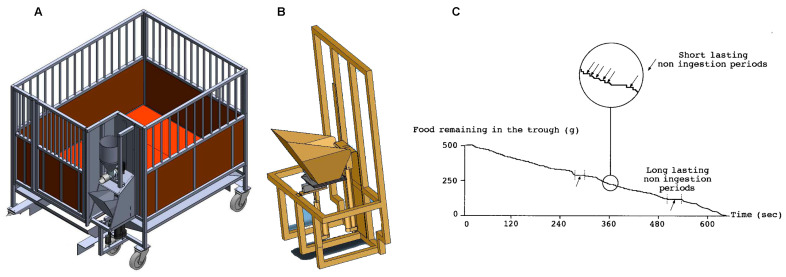
(**A**): Schematic of the individual cage, including the robotic feeder allowing multiple meals per day while analyzing the microstructure of each meal. Note the diet distributor affixed to the top of the feeder, consisting of a large reservoir connected to a worm driven by homemade software allowing precise delivery of the diet. (**B**). Close-up view of the bottom part of the robotic feeder, including the trough, capsize design, and the access door to ensure that the palatability of the diet is identical throughout the day. Three identical feeders can be positioned side by side for meal preference studies. (**C**). Recording of changes in the trough weight during a meal after removal of foraging artifacts. The close-up view illustrates periods of non-ingestion (arrows) during a single meal. Adapted from Ref. [15].

The microstructure of a meal can be obtained readily in individually housed pigs with dedicated robotic feeders (Figure 1). In its most simple design, the device consists of a weight-sensitive sensor attached to the bottom of the trough [14]. Several issues were critical in acquiring and analyzing data recorded from these devices. While constant access to the trough was initially thought to be preferable, this proved not to be the case for several reasons. First, during the within-meal foraging, the pig secretes a large quantity of saliva that, after mixing with the pellets, renders the meal residues far less attractive. This reduction in palatability of the diet is almost impossible to quantify but is intuitively likely to compromise the assessment of satiation. Second, meaningful information from the time interval between two consecutive meals cannot be obtained, which is unfortunately not an index of satiety but rather a reflection of the boredom of the animal. These limitations were overcome by the incorporation of several modifications controlling the access to the trough using a vertical hatch and allowing the remains of the meal to be removed immediately before the initiation of a new meal by capsizing the trough. These improvements allowed the delivery of several meals per day with fresh pellets, accordingly, of stable palatability. Using dedicated algorithms, the rate and duration of meal ingestion, and the number of eating bouts per meal can be extracted from the continuous measurement of the weight of the trough after the detection and removal of foraging artifacts [15,16]. The incorporation of several devices side-by-side (Figure 1 B), and different diets, could be used to assess diet preferences [9,17].

The difficulty in assessing satiety in animal models leads us and others to investigate potential biological proxies. Probably the most promising of these arises from functional neuroimaging methods that are adaptable to the porcine model given that a suitable three-dimensional atlas of the brain is available [8] and integrated into the adequate neuro-imaging tools [18]. In humans, fMRI and ^15^O water PET rely on the hemodynamic response with a time resolution of several seconds. They are, accordingly, well suited to investigate satiation [19]. In animal models, these methods, particularly as anesthesia is mandatory for imaging, do not convey additional value. In contrast, SPECT (single photon emission tomography) after ^99m^Tc-HMPAO administration can characterize brain activation, in the conscious pig, because (i) the penetration of the radioactive molecule is proportional to brain micro vascularization and (ii) it is possible to temporally disconnect the imaging and the stimulus. HMPAO, after its intravenous administration, crosses the brain-blood barrier freely as a lipophilic molecule and penetrates the neuron where bioconversion is achieved, rendering the molecule lipophobic thereby impairing its retro diffusion to the interstitial space [20]. Since this process occurs within 60 s, the distribution of the radioactivity represents a “snap-shoot” of the brain activity that persists for about two hours irrespective of the events occurring after HMPAO injection [21]. This period enables the performance of SPECT imaging in an anesthetized animal without altering the distribution of the radioactivity. The time resolution of the method has been proven to be useful in evaluating satiety during gastric distension [22] and chronic vagal stimulation [23].

While the farm pig represents an easily accessible experimental animal, it is suboptimal since the animal weight is above 200 kg once adult. On the contrary, the miniature pig model allows experiments in adult obese animals with a body weight less than 100 kg. Several western diets with or without fructose have been used, in the miniature pig, to mimic the human metabolic syndrome. However, these dietary interventions were unable to reproduce the actual metabolic syndrome observed in humans—chronic hyperglycemia and hepatic steatosis [24,25]. Furthermore, in the miniature pig and more significantly in the Gottingen breed, the distribution of western diet ad libitum results in alternating periods of hyperphagia and voluntary starvation that extend over a couple of days, resulting in the impossibility to investigate experimentally induced changes in food intake [23]. The most common solution used to overcome this behavior is to supply a limited yet above the dietary requirement both in energy density (about 4000 instead of 2200 kcal per kg of diet) and volume (288 kcal per kg of body weight^0.75^, i.e., 150% of the energy requirement). Nevertheless, the surprising resistance of the obese pig to type 2 diabetes is puzzling [26]. Indeed, the fasting plasma glucose is always less than 7 mmol/L, while the insulin sensitivity was less than 3 dL/kg.min/μU/mL* 1 × 10^−3^ for more than a month. Furthermore, since the obese pig does not present polydipsia or polyuria, the hallmark signs of diabetes, the threshold used to declare diabetes in humans is probably adequate for the pig [27]. On the contrary, the significant insulin resistance and moderate hyperglycemia suggest that the obese pig behaves as a permanent pre-diabetic. This concept is further supported by creating actual diabetes in the obese miniature pig after the additional administration of a small dose of streptozotocin that, alone, is unable to be effective in lean animals [12].

## 3. Gastric Emptying and Meal Distribution

**Figure 2:**
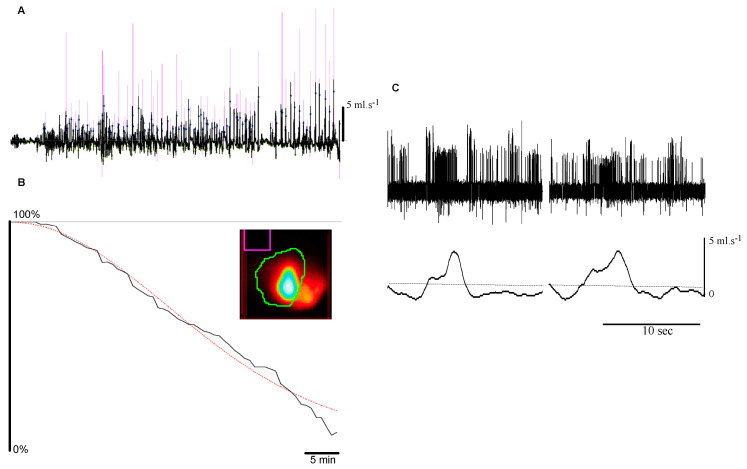
Pulsatile (i.e., “second-by-second”) emptying and vagal information from duodenal vagal receptors sensitive to the stroke volume of individual pulses. (**A**). Transpyloric flow recording (black line) after a 500 mL glucose 10% meal, with detected pulses (dots) and associated stroke volume (proportional to the pink vertical lines). (**B**). Related “overall” emptying measured by scintigraphy of the same meal labeled with 30 MBq of ^99m^Tc-DTPA. The red dotted line represents the power exponential fit of the residual counts per pixel present in the region of interest delineating the stomach (in green on the maximal intensity projection insert). (**C**). Concomitant recording of duodenal afferent neuronal activity with transpyloric passage of the fluid in anesthetized pig. Note the increased frequency of action potentials during the positive flow. Duodenal pressure at the level of the receptive field is constant (not shown).

In humans, the Adelaide team [28] and others [29] have demonstrated that the volume, and the physical characteristics of a meal are critical determinants of satiation. In the pig model, gastric emptying has been poorly investigated by noninvasive methods considered to be the “state of the art” in humans, particularly scintigraphic imaging [30]. Gastric cannulae with or without aspiration of gastric contents have been frequently used in the past and represent an inadequate approach for emptying estimation because of unavoidable numerous artifacts, low temporal resolution, and over-estimation of the emptying rate due to suppression of the gastroduodenal pressure gradient [31]. We pioneered a direct scintigraphic approach in conscious pigs (Figure 2 B), mimicking the method used and validated in humans and employed widely for both clinical and research purposes [32].

Meal labeling has to be partially adapted to the porcine regimen that is at least comparable to humans due to its omnivorous status. As in humans, liquid test meals should be caloric, since water or isotonic saline empties very rapidly (half emptying time less than 15 min). A 500 mL-10% glucose meal [33] is readily ingested by, even poorly compliant, animals. The choices for solid and/or semi-solid meals are theoretically wider. Three test meals are associated with an almost equal quality of binding between the radioactive molecule and the meal constituents: scrambled eggs, grounded beef, and porridge. Of these, porridge is close to the typical cereal-based food for pigs [34]. Furthermore, once reduced into fine particles, it is possible to substitute oats with the animal’s actual feed. Moreover, the half emptying time of this meal is around 2 h, which is compatible with the maximum duration for a pig to stay quietly in a sling frame. Grounded beef is arguably the optimal solid test meal for humans but, in pigs, large inter- and intraindividual variations in the emptying profile are observed with this meal [32], as it is with scrambled egg, which is usually widely used for clinical measurement of gastric emptying in humans.

A limiting factor, in pigs, for correct interpretation of scintigraphic gastric emptying data relates to the absence of readily available analysis software—those designed for use in humans are inadequate. This reflects several issues relating to animal behavior and its anatomical peculiarities. First, lateral imaging correction used to cancel the non-planar anatomy of the stomach is, not surprisingly, mandatory. Indeed, pigs do not tolerate being squeezed between a double head gamma camera, an approach to reduce the planar error. Second, motion generates major artifacts, which is a significant issue, as measurements of solid emptying requiring more prolonged data acquisition. These pitfalls can be addressed satisfactorily using dedicated software integrating depth correction and blur-detection image shifts and/or removal [35,36].

## 4. Gastric signals

### 4.1. Acute Gastric Distension

Several lines of evidence suggest that short-term control of food intake is related to the arrival and storage of the meal in the proximal part of the stomach [37]. Indeed, the experimental proxy of these events e.g., acute balloon distension suppresses food intake [38]. However, data in humans are inconclusive—a 400 mL balloon occupying 30% of the stomach failed to trigger a significant reduction in food intake [39,40,41]. Therefore, it remains unclear whether, if so, and to what extent the mechanical induced signals of gastric fundic and antral distension alter food intake within the meal time-frame. The gastric barostat overcomes the main limitation of a fixed volume distension because it maintains a constant gastric pressure despite the fundic relaxation induced by acute gastric distension [42]. Pressures equal or above 11 mmHg were found to increase meal duration, while volume distensions did not affect feeding behavior irrespective of the gastric bag volume [15]. The wall tension changes are likely to account for these differences, which also explains the discrepancies observed by others using only volume distension.

a. Vagal afferents during gastric distention

At the cervical level, the vagal trunk in the pig exhibits sufficient anatomical separation between its afferent and efferent branches [43] to allow recording of afferent vagal activity using extracellular electrodes during gastric distension. Furthermore, the distance between the recording site and the location of the stimulation is sufficient to avoid motion artifacts that could impair the quality of the single afferent recording [44]. These advantages overcome the difficulties of separating fiber bundles dispersed in a dense connective tissue unlike the scattered lose connective tissue in the cat or the rat. We capitalized on these assets to investigate the single neuron afferent response to gastric distension compared to isovolumetric and barostatic gastric distention in the pig [45]. Surprisingly, the observed increase in spiking activity was the opposite to that expected based on our previous experiment in conscious pigs [15]. Irrespective of distension pressure, volume distension was shown to be the most effective stimulus to increase spiking activity. For the largest volume distensions, there was no significant difference between volume and pressure distentions. Furthermore, some receptors sensitive to volume distension were quiescent during pressure distensions. The observed absence of response for some receptors, while a sustained elevation in pressure was maintained, suggests that, similar to “baro” receptors in the cardiovascular system, circumferential strain might be required to activate these receptors. This possibility has also been suggested based on in vitro recordings using rat explants [46,47]. Taken together, our observations and those obtained from in vitro experiments in rats argue strongly against the often-reported claim that gastric distention activates vagal afferent mechanoreceptors in a dose-dependent manner [48]. This implies that some sort of preconditioning of the distension-related information occurs at the periphery in turn alleviating the processing burden of the primary vagal integrating centers.

b. Central processing

**Figure 3:**
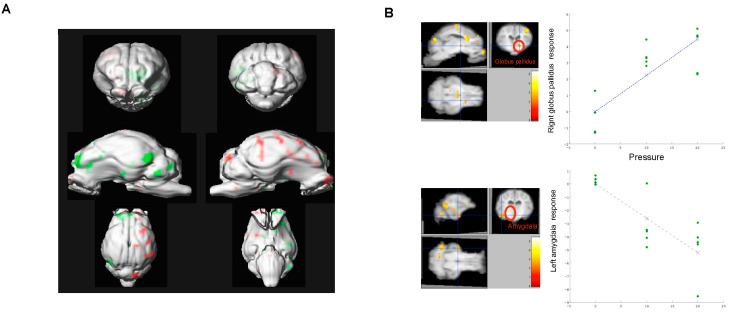
Brain activity during acute gastric distension measured by ^99m^Tc HMPAO uptake in conscious growing pigs. (**A**). Statistical parameter mapping analysis of activated (green) and de-activated (red) brain areas during acute mild gastric distension versus no distension. (**B**). Relationship between gastric pressure and hemodynamic response in activated (Globus palidus) and de-activated areas (Amygdala).

Ultimately, following primary integration on the dorsal vagal nuclei, information encoding distention is processed by secondary brain networks. The engagement of these networks in humans has been studied by either PET ^15^O or fMRI. In these studies, a balloon was orally placed in the stomach, and its volume quickly enlarged to elicit fullness or pain [49,50]. When inflation of the balloon is more gradual, the temporal limitation of the BOLD signal used for fMRI requires that the balloon is inflated and deflated every minute, a situation that mimics post-prandial distention only remotely [51]. Furthermore, the presence in the throat of a gastric tube connecting the distension device to the balloon potentially represents a substantial confounder exacerbating emotional salience [52]. Taking advantage of the precise determination of the maximal pressure achieved, and the temporal pressure changes, during a meal, we have recreated, in the conscious pig, the strains and stresses occurring at the gastric wall during a meal. Furthermore, the barostat bag was inserted through a permanent, surgically prepared access to the gastric lumen. Finally, brain tissue perfusion changes during the entire cycle associated with the virtual meal were detected using SPECT-HMPAO imaging—a time-independent representation of brain activation [22]. The major regions located along vagal-related ascending pathways were activated: brainstem, periaqueductal grey, thalamus, and olfactory bulb. Unrelated vagal regions were also engaged, such as the globus pallidus and the hippocampus/amygdala, suggesting that the reward network might also be involved during mild gastric (Figure 3) [53]. The importance of the reward network engagement through physical stimulation may be of particular relevance to the success of gastric bypass surgery in obese individuals [54,55].

The contribution of spinal afferents to the gastric afferent information is classically considered to be negligible, especially at low pressures, such as those occurring during a meal [56]. Unlike humans, the pig tolerates vagotomy without drainage [33] allowing the investigation of central processing of vagal vs. spinal gastric information. The brain areas engaged during distension changed following vagotomy [57]. As expected, brain structures related to vagal processing were modulated only before vagotomy, e.g., pons, thalamus, prefrontal, and amygdala-hippocampus cortices. However, after vagotomy, the activity of several brain areas still correlated with fundic pressure e.g., colliculus superior, medulla, amygdala. These observations established both that the reward network is involved only by vagal afferent information occurring during gastric distension and that proximal gastric distension has the capacity to activate brain areas such as the amygdala when the vagal pathways have been severed, consistent with the concept of a significant spinal contribution even at a low distending pressure.

### 4.2. Chronic Gastric Distension

Permanent distension of the stomach with water or an air-filled balloon aimed to be an alternative therapy to bariatric surgery. These devices are in many cases well tolerated and relatively easy to insert and remove by endoscopy [58] but their efficacy in reducing weight and increasing satiety remains uncertain [59]. Using a miniature obese pig model, we have demonstrated that an air-filled balloon, after a couple of weeks, instead of reducing gastric volume, increased it by about one third [60]. This reflected an increase in fundic compliance measured by the pressure-volume slope during step-wise barostatic distension. Furthermore, we also observed a reduction in gastric emptying of a porridge meal, which is probably a consequence of the decreased gastric tone. These effects may well contribute to the limited efficacy of balloon therapy in obese patients.

The potential for a difference in brain processing related to the chronic presence of an intragastric balloon, especially in comparison with those occurring as a result of acute gastric distension, was also investigated. Unfortunately, it is impossible to standardize intragastric pressure in these circumstances since changes in pressure within the chronic balloon are not readily accessible. Nevertheless, the intragastric balloon (chronic distension) was associated with activation of the olfactory bulb, prefrontral cortex, nucleus accumbens, thalamus, posterior amygdala, and pons [61]. These brain structures were also engaged during acute distension, except the prefrontal cortex and the nucleus accumbens. The divergence between the minor behavioral consequence of chronic gastric distension on food intake, on the one hand, and the substantial reward network engagement, on the other, was unexpected. Furthermore, the activations of the prefrontal cortex and the nucleus accumbens by acute distension might only reflect the importance of these structures in hedonistic aspects. Indeed, the human prefrontal cortex and nucleus accumbens are specifically reactive to pleasant, rewarding stimuli and are not engaged by unpleasant stimuli [62].

## 5. Intestinal Signals

The importance of the pig model to current understanding of the role of intestinal nutrients to appetite control has been reviewed [63], particularly in relation to peripheral nutrient-sensing [64]. Recently, we showed that mimicking peripheral chemo-sensing with artificial sweeteners has a global impact on insulin sensitivity far beyond appetite control [65].

### 5.1. Transpyloric Flow

In several animal models, including the pig, gastric emptying is predominantly pulsatile rather than continuous, supplying nutrients as a series of gushes in the duodenum (Figure 2A) [66]. In these models, the flow pulses last about 4 s every 15 s, resulting in a stroke volume of 0.9 mL per pulse. The forward to backward flow ratio is about 4:1 and this has been confirmed in humans using Doppler ultrasonography [67]. The pulsatile arrival of nutrients in the intestine was not evident to gastric emptying measurements in humans using either scintigraphy or stable isotope breath test because of their much lower temporal resolution, but has potential consequences. The sudden passage of fluid results in irregular arrival of a significant volume of fluid within the narrow proximal duodenum, probably activating low threshold duodenal vagal mechanoreceptors (Figure 2C). Indeed, there is a linear relationship between the interspike distribution of duodenal vagal afferents and the stroke volume, but not the duration or the peak flow of flow pulses [68]. Furthermore, neither the circumferential strain recorded by a strain gauge affixed on the serosa nor the fluid velocity were related to the firing of receptors, suggesting that duodenal mechanoreceptors primary detect the stroke volume of the pulses. Despite evidence for vagal afferent coding, the contribution of pulsatile transpyloric flow to appetite control appears unimportant. Indeed, in humans, antropyloroduodenal pressures, plasma CCK concentration, and appetite are not modified by pulsatile versus constant infusion of lipid into the duodenum [69]. The situation might be different in obese after gastric bariatric surgery since it results in a much more rapid emptying [70] probably as a consequence of flow pulses of larger stroke volume. Unfortunately, experimental data is missing to support this hypothesis since Roux en Y surgery in the porcine model increases paradoxical glucose metabolism [71] and accordingly no attempt to record transpyloric flow, in these conditions, have been done in the porcine model.

### 5.2. Jejunal vs. Portal Signals

Vagally mediated appetite control is dependent on the integration of glucose-sensing mechanisms located in the brain, portal vein, and intestine [72]. The impact of obesity and insulin resistance on brain glucose sensing has been investigated extensively [73], but there is much less information about the hepato-portal sensor [74] probably, in part, because it is much less accessible.

**Figure 4:**
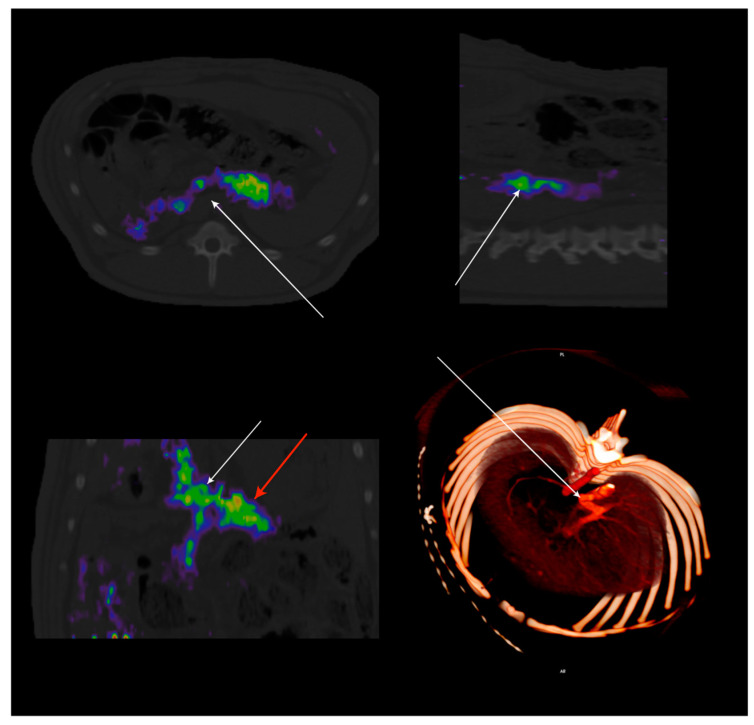
Hybrid PET-CT imaging of GLP-1r distribution along the portal vein using ^68^Ga-DO3A-Exendin-4 (0.2MBq/Kg) was administered IV in adult miniature pigs. Each color pixel is coded to represent the binding potential of GLP-1r. Note the localization of the GLP-1r along the portal vein immediately before its entrance into the liver. The density of GLP-1r parallels that of the portal glucose sensor since the GLP-1r is critical for vagal signal transduction. Radioactive binding is not evident in obese adult miniature pigs due to the suppression of the glucose sensor. Adapted from Ref. [79].

There is indirect evidence for a neuronal circuit responsible for a regulatory response to portal hyperglycemia [75]. Unfortunately, there is a lack of direct confirmation of portal sensitive neurons, reflecting the sparsity of the portal innervation [4]. Nevertheless, it is clear that glucagon-like peptide-1 receptor (GLP-1r) is critical to portal vagally mediated glucose sensing [56,76]. We identified, in lean and obese mini-pigs, GLP-1-dependent portal glucose signaling, in vivo, using a novel ^68^Ga labeled GLP-1r positron-emitting probe [77] that provided a quantitative in situ tridimensional representation of the portal sensor [78] (Figure 4). We also used this as a map for single-neuron electrophysiology driven by image-based abdominal navigation. In insulin-resistant animals, portal vagal afferents failed to inhibit their spiking activity during glucose infusion, a GLP-1r-dependent function. The importance of a reduction in portal GLP-1r binding potential, particularly between the splenic vein and the liver entrance, was further demonstrated by the suppression of the glucose effect on the afferent by pharmacological inhibition of the GLP-1r, in lean animals only. Accordingly, in the pig, obesity-induced insulin resistance leads to functional portal denervation with marked suppression of vagal sensitivity to portal glucose. The latter appears to be the consequence of a reduction in the density of GLP-1r, as indicated by diminished GLP-1r binding potential in obese insulin-resistant animals [79]. The concept of a functional denervation at the portal level suggests that it might be possible to restore pharmacologically the portal glucose sensor in obese insulin-resistant patient through the expression of GLP-1r.

Despite the importance of the portal sensor for glucose detection, in the pig, duodenal and portal glucose infusions were equally potent in reducing food intake [80]. Both duodenal and portal glucose infusions activated the dorsolateral prefrontal cortex and primary somatosensory cortex. Duodenal glucose infusion also induced activation of the prepyriform area, orbito-frontal cortex, caudate, and putamen [81]. This comparable effectiveness suggests that the intestinal vagal signal is poorly integrated centrally and/or is not essential to the central response to intestinal glucose, particularly as the glycemic response is also identical. Nevertheless, the substantial difference in brain matrix observed for portal vs. duodenal infusion with a comparable behavioral outcome is surprising and might reflect the limitation of SPECT-HMPAO imaging. Indeed, like fMRI, SPECT-HMPAO is at best semi-quantitative with a mandatory normalization using either the entire brain tissue or the cerebellum as a reference. This step, which is not required for PET when an arterial input function is simultaneously acquired, has the potential to generate artifactual activation, especially for low significance statistical threshold [82].

## 6. Mimicking Abdominal Afferent Vagal Signaling

### 6.1. Importance of Vagal Afferents for Appetite

Despite the intricacies of the digestive vagal information, several attempts have been made to manipulate this information using electrical nerve stimulation. Vagotomy has historically been a therapy for peptic ulcers, where some obese patients experienced weight loss [83]. Based on these observations and trials of bilateral vagotomy as a treatment for obesity [84,85], even though the results were mitigated-a device aimed at inducing vagal blockade has been developed to generate weight loss. Not surprisingly, reflecting the extreme intricacy, even in vitro, of achieving effective nerve blockade, the therapy proved to be unsuccessful albeit safe in obese humans [86]. In contrast, our data in obese pigs [14,87] together with the important observations from murine models [88,89,90] demonstrated that vagal signals are attenuated in obese models suggesting that therapy must be based on stimulation rather than inhibition of the vagal signal. However, mimicking abdominal afferent vagal signaling using current pulses applied on the vagus represents only one option for the restoration of the vagal traffic between the gut and the brain. Alternatives solutions such as pharmacological modulation of the vagal sensors peripherally or modifications of the primary and secondary brain networks related to vagal inputs represent equally attractive, non-mutually exclusive, options for obesity treatment.

### 6.2. Vagal Afferents Plasticity

Vagal afferents are inherently plastic, and experimental evidence obtained in rats and mice shows that they can change the synaptic number, neuronal excitability, and neuropeptide expression in response to peripheral stimuli. These have been recently reviewed by de Lartigue and Xu [91]. Nevertheless, the possibility for vagal afferents to switch from expressing anorectic to orexigenic neuropeptides as a consequence of metabolic challenges is still a matter of debate [92]. On the contrary, the mitigation of vagal afferent mechanical sensing during obesity is firmly established [47,93]. However, the functional consequences of peripheral plasticity are still largely putative, mostly because equally important plasticity occurs in secondary projection areas of vagal afferents [94]. The final integration of the peripheral signals is intuitively more complex than the raw addition of central and peripheral plasticity, but the mathematical tools capable of handling these intermingled modulations quantitatively are scarce [95]. Furthermore, the demonstration of these in porcine model is still missing.

### 6.3. Early Outcomes of Abdominal Vagal Stimulation

Together with other groups [96], we initiated chronic vagal stimulation in pigs to investigate bilateral stimulation of the abdominal vagal nerves (VNS). The first attempt to identify the optimal location of the electrodes to suppress food intake was by Laskiewicz et al., in rats [97]. They reported that bilateral VNS is more effective than unilateral vagal stimulation [98]. Using the porcine model, we initially evaluated juxta-abdominal bilateral vagal stimulation in an attempt to minimize adverse cardiovascular effects. This location proved to decrease weight gain, food consumption, and sweet craving in both growing pigs [14] and adult obese minipigs [99]. However, in both normal-weight and obese animals, the reductions in food intake and body weight were modest. Indeed, in both situations, body weight and food intake continued to rise as the animals became older, although the rate at which this occurred was reduced by vagal stimulation. It is likely that the limited effects reflected the infra-optimal stimulation parameters since stimulation was achieved using two separate clinical VNS stimulators that were not working in synchronicity on both vagi. Furthermore, electrical compliance of these generators while sufficient for the small diameter of the human cervical vagal nerve may well have been inappropriate for the larger abdominal vagus of the pig [100]. Nevertheless, while recognizing that the methodology was suboptimal, an essential step was the demonstration that vagal stimulation did not modify gastric emptying in a large animal model [22] suggesting that epigastric fullness and other symptoms related to increased gastric retention are unlikely to occur and/or are not causative for the behavioral effects of vagal stimulation. The early trials in vagal stimulation, while being partly unconclusive, paved the way for more adequate technologies that we developed during the past years, including laparoscopically implantable electrode cuffs suitable for the abdominal vagus, double stage, high compliance, synchronous, current stimulator appropriate for chronic implantation, together with remote wireless monitoring of the changes in electrical impedance of the nerve-electrode complex representative of the interlocking between the cuff and the perineurium [23]. These technologies were applied incrementally in the experiments described in the next paragraphs.

### 6.4. Targeting the Appropriate Neuronal Type

The modest or negligible outcomes of VNS in obese humans may well reflect premature translation from animals to humans without sufficient information about the optimum stimulation profile, current intensity, and more generally therapy characteristics [96]. For example, the concept that the same stimulation current used at the cervical level to alleviate epilepsy would be effective at the abdominal level was naïve. Indeed, at the abdominal level, the majority of vagal neurons located either in the dorsal or ventral vagal trunks are small diameter myelinated and un-myelinated neurons, i.e., Adelta or C type [101]. Therefore, large current pulses are needed to depolarize the axon membrane and generate an action potential-more than 20 mA may be needed [102]. While they proved to be effective acutely, the use of these currents would be unrealistic in a chronic implant, since they generate damage to the electrode and the biological tissues [100]. In anesthetized animals, an innovative stimulation profile (named pulson) applied bilaterally on both abdominal vagal trunks can trigger action potentials in small-diameter neurons (C and Adelta types) [16]. The pulson profile is composed in a short series of very high frequency pulses (>100 Hz) that individually cannot depolarize the neuron. In conscious animals, this stimulation profile could increase the metabolism of the DVC and that of other brain areas that are primary or secondary projections from the DVC. Pulson stimulation was also able to halve the food intake within two weeks, unlike the classical millisecond stimulation pattern that takes several weeks to reduce the slope only [99]. Since this stimulation pattern required only about one-third of the charge needed for a long-lasting classical pulse to evoke an action potential, it has the potential to be used chronically without altering the integrity of the nerve [17,103,104]. Given its efficacy to reduce food intake, a feature never encounter before, this solution deserves further studies in animal model and in obese humans.

### 6.5. Central Effects of VNS

Regardless of the significant improvements in electrode placement, stimulation scheme, and hardware design in the last five years, early clues to refine stimulation parameters without waiting to observe changes in body weight are needed. It is probable that brain-imaging methods and the computational model of VNS [100,105] may be useful. Several studies have investigated the effect of left cervical VNS on brain function using PET and fMRI imaging both in animals and humans. In epileptic and depressive patients, cervical VNS induced a gradual brain response involving a change in dorsolateral prefrontal/cingulate cortical activities followed by dopaminergic activation of the limbic system [106] or limbic-connected structures [107]. A widespread engagement involving several functional brain networks [108] has also been identified.

**Figure 5:**
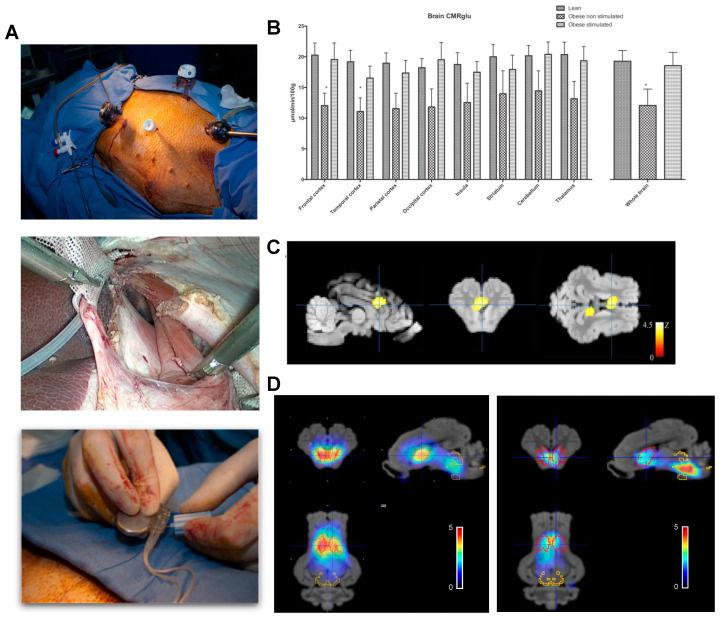
Brain metabolism and SERT/DAT expression in adult miniature pigs after chronic abdominal vagal stimulation. (**A**). Laparoscopic access to the abdominal vagus at the level of the lower esophageal sphincter in the obese miniature pig. Two cuffs with electrode pairs Pt-Ir are located around each vagal nerve after careful dissection and subsequent closure of the esophageal groove. The electrode leads were connected to purpose made, double current high compliance channels, neurostimulator that was implanted in a subcutaneous pocket [109]. (**B**). Quantitative changes in brain glucose uptake after several weeks of chronic vagal stimulation in lean and obese animals showing restoration of obesity-related impaired glucose metabolism by VNS. (**C**). Voxel-based statistical parametric mapping analysis showing the differences in glucose metabolism between the obese non-stimulated and obese-stimulated groups. The image was centered the dorsal anterior cingular cortex, which was the region most markedly affected by stimulation. (**D**). Pixel-wise modeled SPECT dynamic image after administration of ^123^I ioflupane showing the binding potential of DAT/SERT overlaid on the MRI template. Red VOIs correspond to DAT-rich areas, whereas yellow VOIs represent SERT-rich areas. The left panel represents sham, whereas the right panel shows vagal stimulated obese miniature pigs. Adapted from Refs. [17,104].

We reported the only quantitative brain map of glucose metabolism induced by abdominal VNS in obese preclinical pig models (Figure 5). Using this unbiased analysis, statistical parameter mapping performed on quantitative glucose-uptake images showed that brain glucose uptake was increased in the stimulated animal but only in a limited number of brain areas [109] including the periaqueducal grey, the thalamic and hypothalamic areas, and part of the amygdala and the insular cortex (Figure 5C). More importantly, enhanced brain connectivity in several regions including the striatum, cingulate, insula, thalamus, amygdala, hippocampus, and mid-brain were identified. These changes were associated together with profound alterations in DAT and SERT binding potentials. DAT binding potential was decreased markedly in the striatum while SERT binding potential was doubled in the mid-brain [17], (Figure 5D). These changes may be fundamental to the reduced food intake induced by VNS since the mesolimbic dopamine reward system is central to the regulation of eating behavior [110], and dopamine receptors availability has been reported to be reduced in morbidly obese individuals [111].

### 6.6. VNS Improves Insulin Sensitivity

Animal studies provide persuasive evidence that acute vagal stimulation increases fasting insulin release from the pancreas [112]. In contrast, the effect of chronic vagal stimulation on insulin sensitivity has received much less attention. One study in Zucker rats suggested that chronic vagal stimulation may up-regulate insulin receptor expression in the brain, liver, and skeletal muscle [113]. In obese pigs, chronic bilateral vagal stimulation can restore fasting glucose metabolism. This effect is evident at the whole-body level and in the brain, the liver, and the skeletal muscle and is associated with reductions in fasting glucose and insulin. The observed changes in glucose metabolism in the brain were also area-specific, with particular involvement of an amygdala-cingulate network and, more generally, several parts of the limbic system. The importance of the cingulate in insulin secretion is of specific interest, since electrical stimulation of the dorsal cingulate cortex in the dog suppresses insulin secretion in response to an intravenous glucose load [114]. Similarly, there is evidence that the cingulate cortices are involved in the brain response to the GLP1 agonist, exenatide [115], which may also improve insulin sensitivity. While chronic bilateral vagal stimulation is exceptionally successful in the restoration of insulin sensitivity, long-term efficacy on primary outcomes remains to be established. A further challenge is to translate favorable trial outcomes to a real-world setting.

## 7. Conclusions

In conclusion, the porcine model has provided unique data on the peripheral modulation of the vagal afferent information in lean and obese animals. Its size was adequate to translate recording tools from clinical to research setting while being possible to access invasively vagal activity—an ethical issue in human research. Furthermore, it has been demonstrated that the engagement of primary and secondary vagal projection brain areas was extremely sensitive to peripheral sensing of the vagal afferent and its modulation by diet and pathophysiological conditions such as obesity. This unpredicted behavior, uncorrelated with changes in satiation or satiety, militates for a stringent threshold during the analysis of brain activation maps within the scope of appetite control. It also points out the absolute requirement for quantitative analysis of these maps. Finally, the similarities of the abdominal vagus between the pig and the human, a condition fundamentally different from that observed in the murine models, constitute a driving force towards innovative therapy tools engulfing developments in material physics, electronics, and radiochemistry.

## Data Availability

No new data were created or analyzed in this study. Data sharing is not applicable to this article.

## Abbreviations

BOLD	Blood-oxygen-level dependent
DAT	Dopamine active transporter
DVC	Dorsal vagal complex
fMRI	functional magnetic resonance imaging
GLP-1r	Glucagon like peptide-1 receptor
HMPAO	hexa-methyl-propyl-amineoxime
PET	Positron emission tomography
SPECT	Single photon emission computed tomography
SERT	sodium-dependent serotonin transporter
VNS	vagal nerve stimulation
VOI	volume of interest
